# Supplementary material for: The Knowledge Connector decision support system for multiomics-based precision oncology
Source: Nat Commun. 2026 Jan 19;17:742. doi: 10.1038/s41467-026-68333-3 (PMC12820158; doi:10.1038/s41467-026-68333-3)
Supplement: Supplementary file 1 — Supplementary Information [file 41467_2026_68333_MOESM1_ESM.pdf]

Supplementary Information

The Knowledge Connector decision support system for multiomics-based precision oncology

Daniel Hübschmann[1,2,3,4,5,*,✉], Simon Kreutzfeldt[3,6,*], Benjamin Roth[7,*], Katrin Glocker[7,8,*], Janine Schoop[7], Lena Oeser[7], Steffen Hausmann[7], Christian Koch[7], Sebastian Uhrig[1], Jennifer Hüllein[1], Barbara Hutter[1], Martina Fröhlich[1], Christoph E. Heilig[6], Maria-Veronica Teleanu[6], Daniel B. Lipka[3,6], Irina A. Kerle[9,10,11], Annika Baude-Müller[6], Katja Beck[6], Christoph Heining[9,10,11], Hanno Glimm[9,10,11], Frank Ückert[12], Alexander Knurr[7,#], Stefan Fröhling[3,5,6,#,✉],Peter Horak[3,6,#,✉]

[1] Computational Oncology Group, Molecular Precision Oncology Program, National Center for Tumor Diseases (NCT), NCT Heidelberg, a partnership between the German Cancer Research Center (DKFZ) and Heidelberg University Hospital, Heidelberg, Germany

[2] Pattern Recognition and Digital Medicine Group, Heidelberg Institute for Stem Cell Technology and Experimental Medicine (HI-STEM), Heidelberg, Germany

[3] German Cancer Consortium (DKTK), DKFZ, Core Center Heidelberg, Heidelberg, Germany

[4] Innovation and Service Unit for Bioinformatics and Precision Medicine, DKFZ, Heidelberg, Germany

[5] Institute of Human Genetics, Heidelberg University Hospital, Heidelberg, Germany

[6] Division of Translational Medical Oncology, DKFZ, and NCT Heidelberg, Heidelberg, Germany

[7] Secondary Use of Data in Oncology Group, Clinical Trial Office, DKFZ, Heidelberg, Germany

[8] Medical Genetics Center, Munich, Germany

[9] Department of Translational Medical Oncology, NCT, NCT/University Cancer Center (NCT/UCC) Dresden, a partnership between DKFZ, Faculty of Medicine and University Hospital Carl Gustav Carus, TUD Dresden University of Technology, and Helmholtz-Zentrum Dresden-Rossendorf (HZDR), Dresden, Germany

[10] Translational Medical Oncology, Faculty of Medicine and University Hospital Carl Gustav Carus, TUD Dresden University of Technology, Dresden, Germany

[11] DKTK, Partner Site Dresden, Dresden, Germany

[12] Institute for Applied Medical Informatics, Medical Center Hamburg-Eppendorf, Hamburg, Germany

[*] These authors contributed equally.

[#] These authors jointly supervised this work.

[✉] Correspondence should be addressed to these authors.

Table of Contents

Supplementary Fig. 1 - 8

Supplementary Tables 1-27

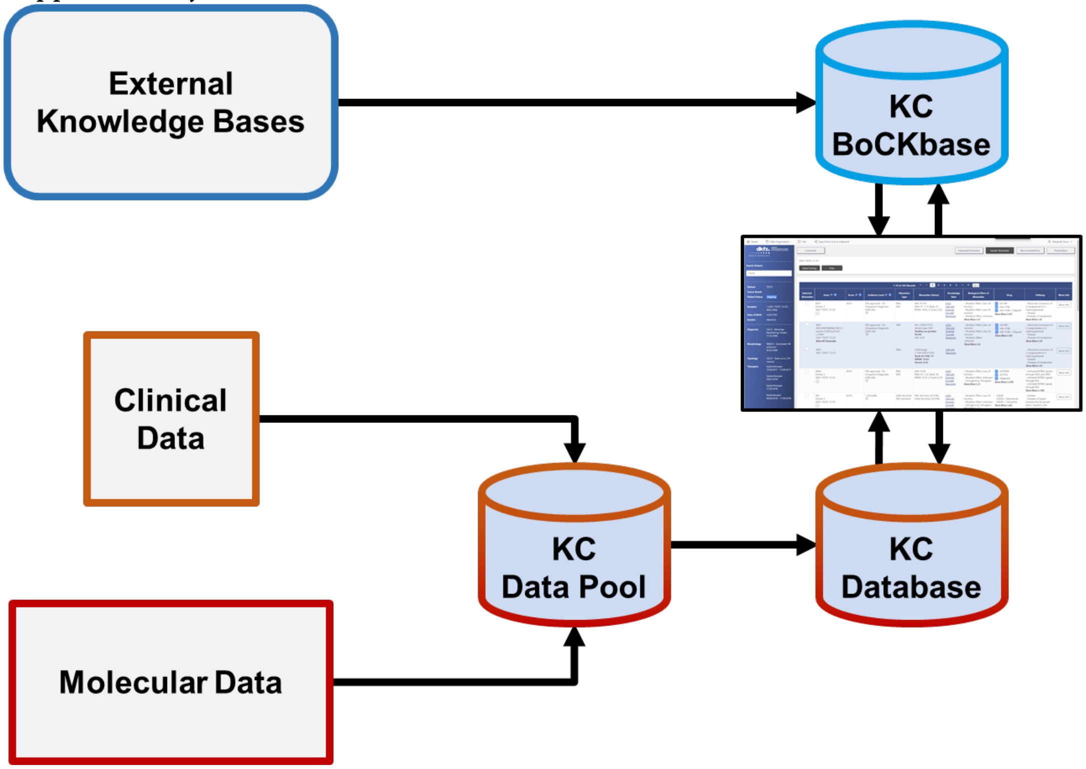

Supplementary Fig. 1: Data flow of the KC.
Data from external knowledge bases are extracted and imported into the BoCKbase. Patient-related clinical and molecular data from EHRs and the bioinformatics workflow are stored in the KC Data Pool in a defined data model, prepared for display in the KC, and transferred to the KC Database. In the KC, the BoCKbase is queried for relevant information based on the patient data.

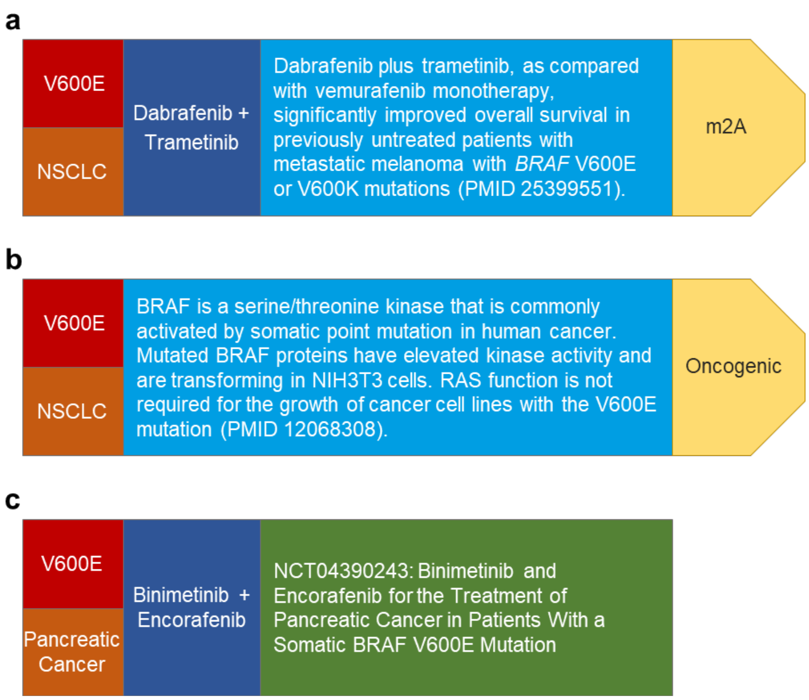

Supplementary Fig. 2: Examples of BoCKs.

a, BoCK linking a BRAF V600E mutation with dabrafenib and trametinib combination therapy in non-small cell lung cancer (NSCLC) and assigning a predictive statement with a specific mEL. **b,** BoCK linking a BRAF V600E mutation with the functional assessment of its oncogenicity in NSCLC. **c,** BoCK combining a BRAF V600E mutation with eligibility for a clinical trial with encorafenib and binimetinib.

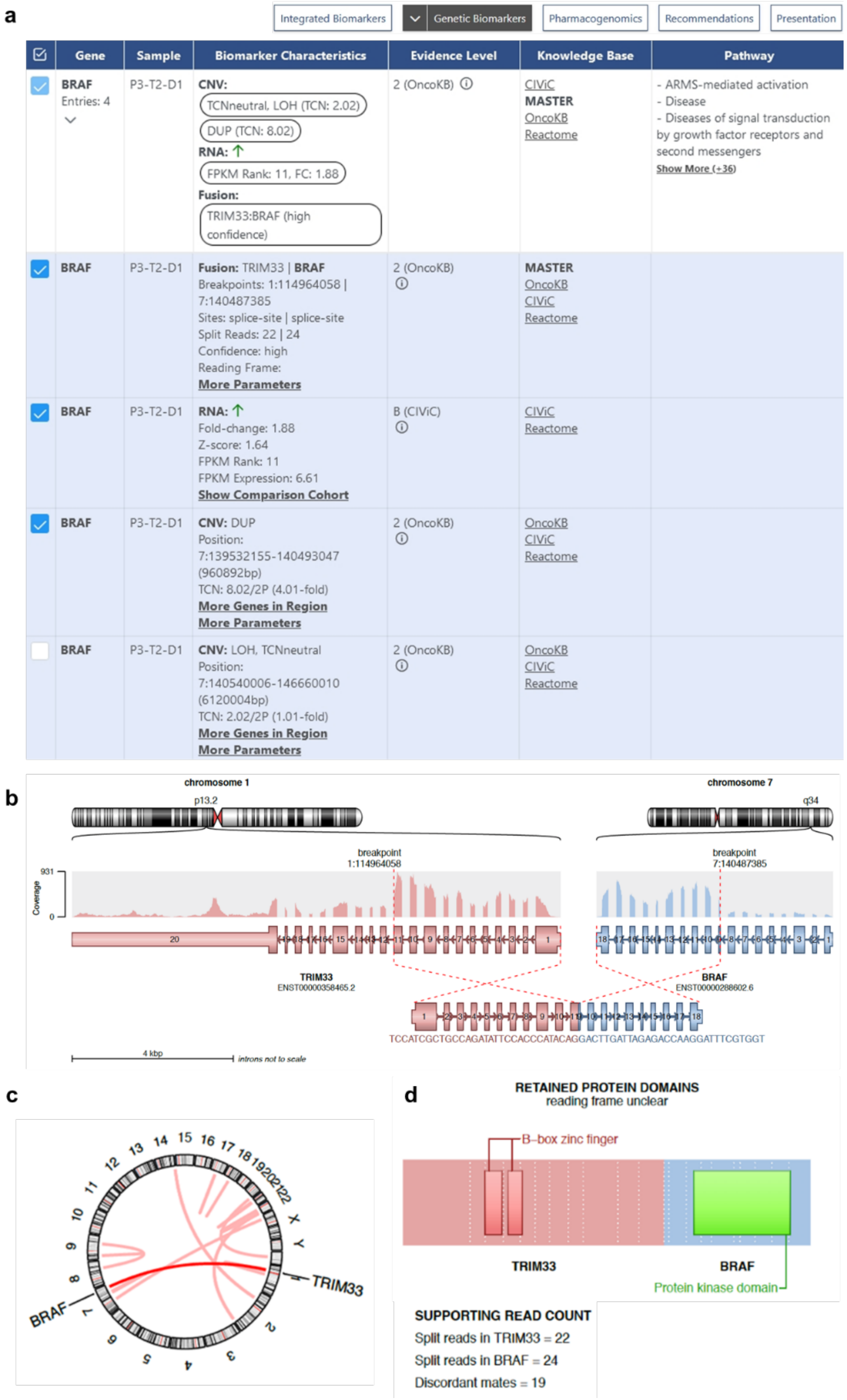

Supplementary Fig. 3: KC display of a TRIM33::BRAF fusion.
a, Screenshot of the "Genetic Biomarkers" panel illustrating the congruent detection of the fusion gene and an overexpressed fusion transcript. **b,** Visualization of the TRIM33::BRAF fusion event by the fusion detection tool Arriba. **c,** Chromosomal position of the TRIM33::BRAF fusion. **d,** Domain structure of the resulting TRIM33::BRAF fusion protein.

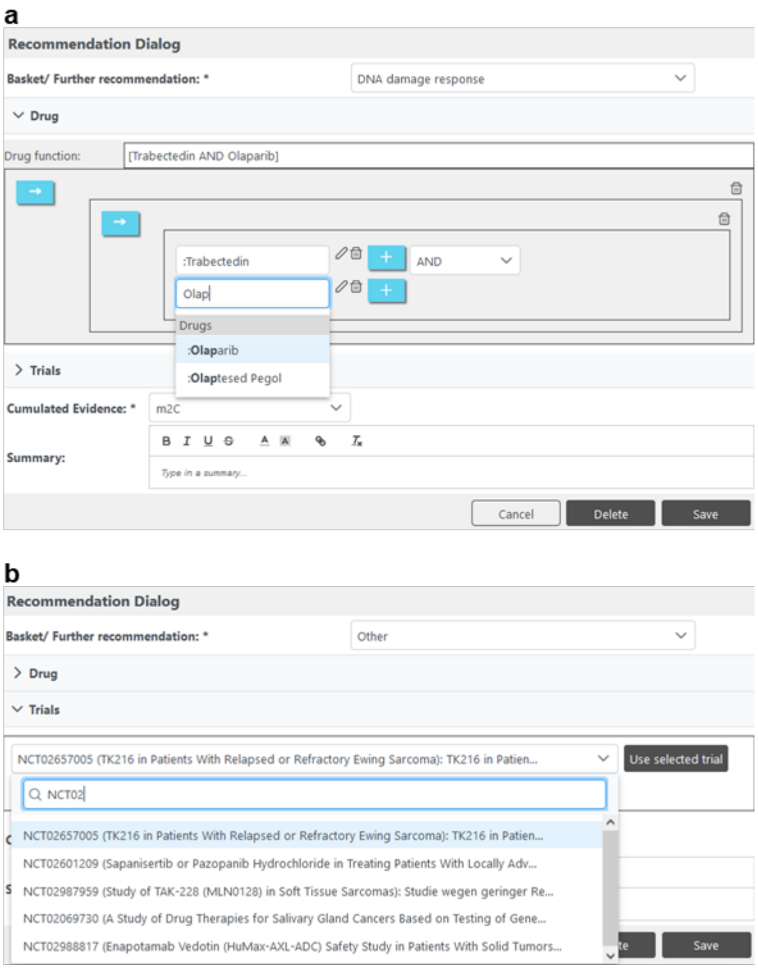

Supplementary Fig. 4: Drug and clinical trial assignment to MTB recommendations.
a, Recommendation dialog illustrating the assignment of single or multiple drugs to individual treatment recommendations. **b,** Recommendation dialog illustrating the search for and assignment of clinical trial BoCKs to individual treatment recommendations.

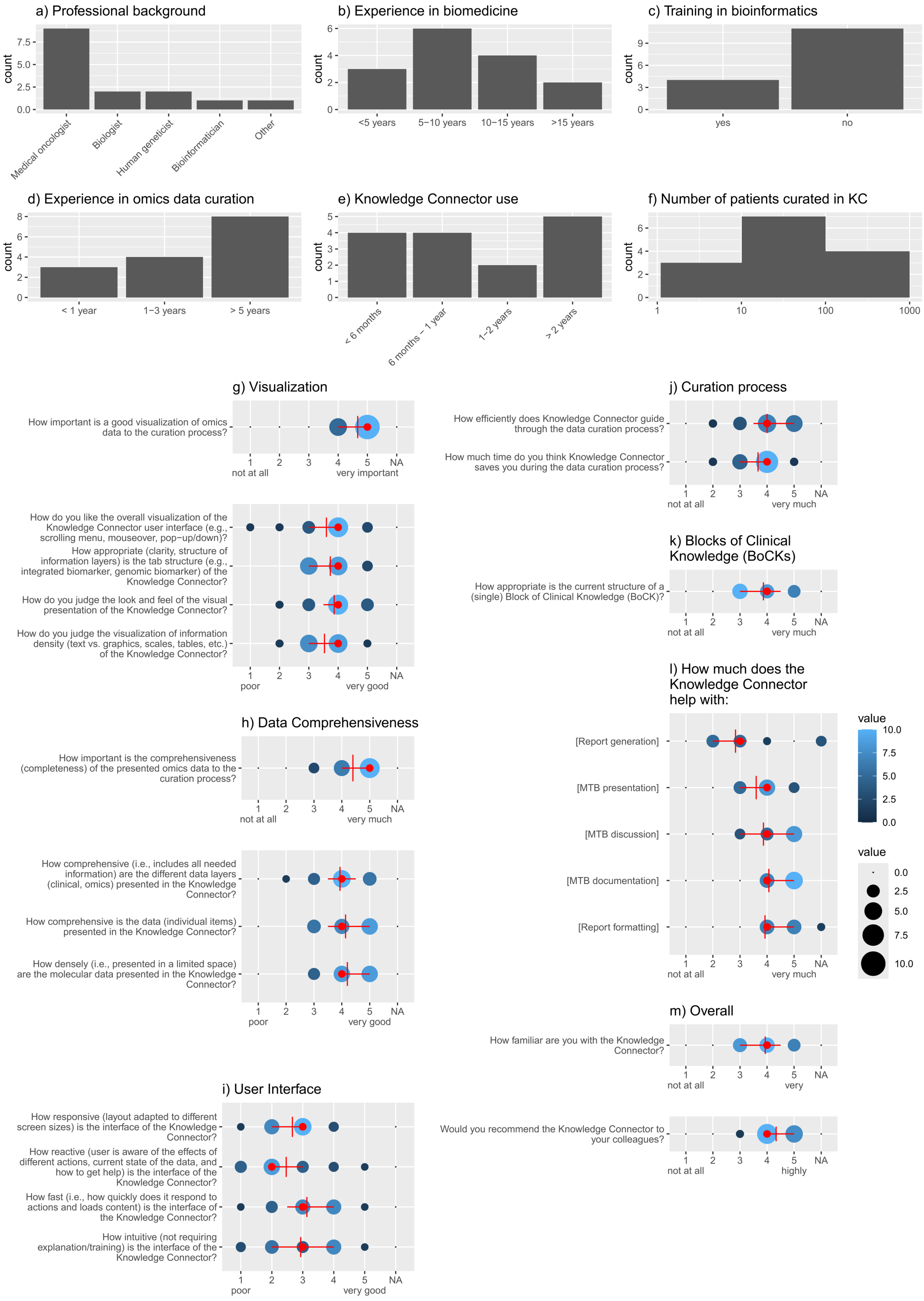

Supplementary Fig. 5: Structured survey results among KC users (n=15).
a, Professional background of respondents. **b**, Experience in biomedicine. **c**, Training in bioinformatics. **d**, Experience with multiomics data curation. **e**, Duration of KC use. **f**, Number of self-reported KC-curated cases (0–10, 10–100, 100–1,000 cases). **g**, Evaluation of visualization features. **h**, Assessment of data comprehensiveness. **i**, Evaluation of the user interface. **j**, Feedback on the curation process. **k**, Evaluation of BoCKs. **l**, Evaluation of the overall workflow. **m**, Overall evaluation of KC adoption.

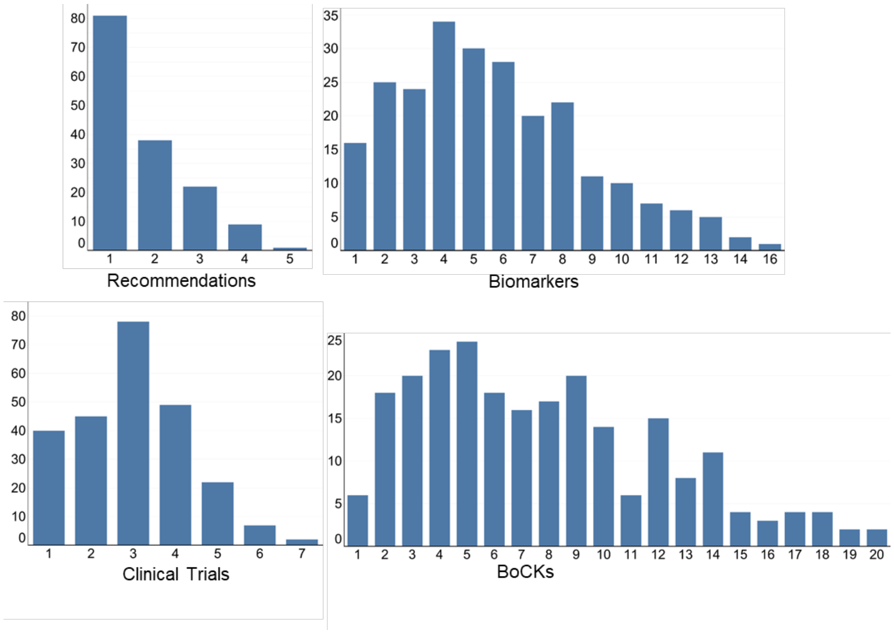

Supplementary Fig. 6: Overview of 268 MTB cases curated using the KC.
Number of therapeutic recommendations (n=243, 90.7%), actionable biomarkers (n=241, 89.9%), clinical trial matches (n=151, 56.3%), and BoCKs (n=235, 87.7%) per MTB case.

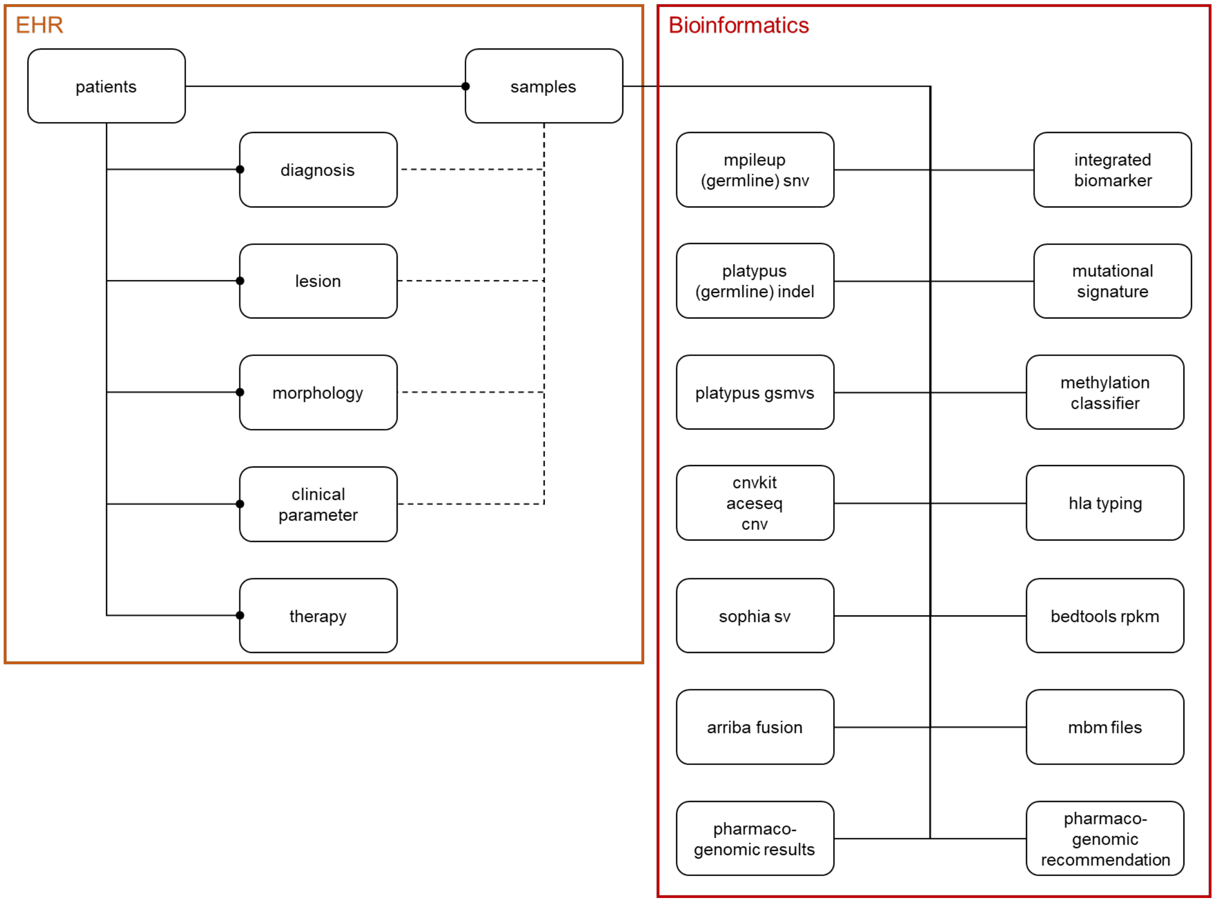

Supplementary Fig. 7: Patient-related data elements stored in a defined data model in the KC Data Pool.
Clinical data from EHRs are linked to individual patients and can optionally be associated with corresponding samples. The bioinformatics workflow provides quality-filtered, annotated molecular data, which are connected to the patient via the sample.

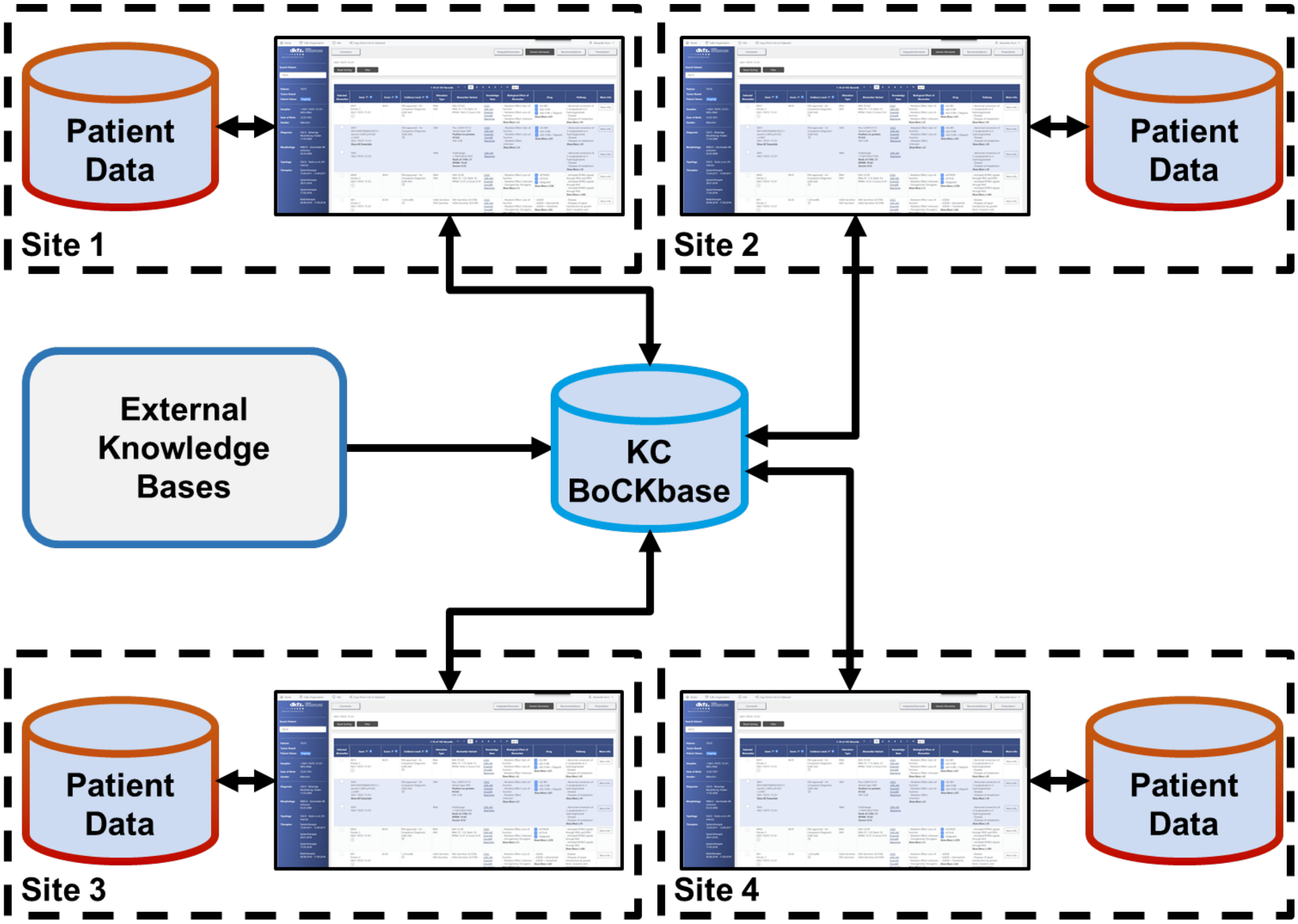

Supplementary Fig. 8: Cross-site data flows of the KC.
By separating the KC BoCKbase from the KC Data Pool and the KC Database, a joint BoCKbase can be created and used by collaborating institutions while the patient data remain at the individual sites.

Supplementary Table 1: Genetic data features of selected decision support tools and knowledge bases

Genetic Data Feature	KC	MO Almanac	CGI	MTB Portal	Sophia DDM[A]	QCI Interpret[A]	MH Guide[A]	OncoKB[B]	CIVIC[B]	CKB[B]
**DNA sequencing data support**	WGS, WES	WES, panel	WGS, WES, panel (VCF input)	Panel	WES, panel	WGS, WES, panel	WGS, WES, panel	N/A	N/A	N/A
**Small variants (SNVs/indels)**	Yes	Yes	Yes	Yes	Yes	Yes	Yes	Yes	Yes	Yes
**Copy number alterations**	Yes	Yes	Yes	Yes	Yes	Yes	Yes (gene level)	Yes	Yes	Yes
**Structural variants**	Yes	Yes	Yes	No	Yes	Yes (if detected)	Yes (if detected)	Yes	Yes	Yes
**RNA sequencing data support**	WTS	WTS (fusions)	No	Yes (panel)	Targeted (fusions)	No	No	N/A	N/A	N/A
**Gene fusions**	Yes	Yes (RNA)	Yes (DNA)	No	Yes	Yes (manual)	Yes (manual)	Yes	Yes	Yes
**Complex biomarkers**	Yes	Yes	No	No	Yes	Yes	Yes	Yes	Yes	Yes (MSI, TMB)
**Pharmacogenomics**	Yes	No	No	No	No	No	No	No	Yes	No
**Germline alterations**	Yes	Yes	No	No	No	No (separate tool)	No (separate tool)	No	No	No

[A] Commercial decision support tool
[B] Knowledge base

Abbreviations: CGI, Cancer Genome Interpreter, https://www.cgiclinics.eu; CIVIC, Clinical Interpretation of Variants in Cancer, https://civicdb.org; CKB, Cancer Knowledgebase, https://ckbhome.jax.org; MH Guide, Molecular Health Guide, https://analysis.mh.guide; MO Almanac, Molecular Oncology Almanac, https://moalmanac.org; MSI, microsatellite instability; MTB Portal, Molecular Tumor Board Portal, https://www.mtbp.org; OnkoKB, Oncology Knowledge Base, https://www.oncokb.org; QCI Interpret, Qiagen Clinical Insight Interpret, https://digitalinsights.qiagen.com; Sophia DDM, Sophia Genetics Data-Driven Medicine, https://www.sophiagenetics.com/sophia-ddm; TMB, tumor mutational burden; VCF, variant call format; WES, whole-exome sequencing; WGS, whole-genome sequencing; WTS, whole-transcriptome sequencing

Supplementary Table 2: Knowledge base features of selected decision support tools and knowledge bases

Knowledge Base Feature	KC	MO Almanac	CGI	MTB Portal	Sophia DDM[A]	QCI Interpret[A]	MH Guide[A]	OncoKB[B]	CIVIC[B]	CKB[B]
**Biomarker identification**	Yes	Yes	Yes	Yes	Yes	Yes	Yes	Yes	Yes	Yes
**Alteration details**	Yes (variant annotation)	Yes (algorithm)	Yes (driver/ passenger)	Yes (variant annotation)	Yes (curated evidence)	Yes (curated evidence)	Yes (curated evidence)	Yes (onco-genicity)	Yes (variant annotation)	Yes (effect)
**Tumor context**	Yes	Yes	Yes	Yes	Yes	Yes	Yes	Yes	Yes	Yes
**Actionability ranking**	Yes	Yes	Yes	Yes	Yes	Yes	Yes	Yes	Yes	Yes
**Drug information**	Yes	Yes	Yes	Yes	Yes	Yes	Yes	Yes	Yes	Yes
**Clinical trial information**	Yes	No	No	No	Yes	Yes	Yes	No	No	Yes

[A] Commercial decision support tool
[B] Knowledge base

Abbreviations: CGI, Cancer Genome Interpreter, https://www.cgiclinics.eu; CIVIC, Clinical Interpretation of Variants in Cancer, https://civicdb.org; CKB, Cancer Knowledgebase, https://ckbhome.jax.org; MH Guide, Molecular Health Guide, https://analysis.mh.guide; MO Almanac, Molecular Oncology Almanac, https://moalmanac.org; MTB Portal, Molecular Tumor Board Portal, https://www.mtbp.org; OnkoKB, Oncology Knowledge Base, https://www.oncokb.org; QCI Interpret, Qiagen Clinical Insight Interpret, https://digitalinsights.qiagen.com; Sophia DDM, Sophia Genetics Data-Driven Medicine, https://www.sophiagenetics.com/sophia-ddm

Supplementary Table 3: Patient data from EHRs

Column	Type	Description
**pid**	varchar(32)	Patient identifier
**gender**	varchar(16)	Gender
**first_name**	varchar(64)	First name
**last_name**	varchar(64)	Last name
**birthdate**	date	Date of birth
**site**	varchar(128)	Enrolling site
**applicant**	varchar(128)	Referring physician

Supplementary Table 4: Diagnostic data from EHRs

Column	Type	Description
**Pid**	varchar(32)	Patient identifier
**Sample**	varchar(64)	Biosample identifier
**diagnosis_type**	varchar(32)	Classification system for diagnosis (e.g., ICD-10)
**diagnosis_value**	varchar(256)	Diagnosis (e.g., C50.1)
**Entity**	varchar(256)	Entity (e.g., non-small cell lung cancer)
**documentation_date**	timestamp	Date of documentation

Supplementary Table 5: Tumor manifestation data from EHRs

Column	Type	Description
**pid**	varchar(32)	Patient identifier
**sample**	varchar(64)	Biosample identifier
**lesion**	varchar(256)	ICD-O-3 topology
**lesion_type**	varchar(256)	Primary tumor or metastasis
**documentation_date**	timestamp	Date of documentation

Supplementary Table 6: Tumor morphology data from EHRs

Column	Type	Description
**pid**	varchar(32)	Patient identifier
**sample**	varchar(64)	Biosample identifier
**morphology_type**	varchar(32)	Classification system for morphology (e.g., ICD-O-3)
**morphology_value**	varchar(256)	Morphology (e.g., 8500/3)
**tumor_content**	varchar(256)	Tumor cell content
**grading**	varchar(256)	Histologic grading
**documentation_date**	timestamp	Date of documentation

Supplementary Table 7: Clinical parameters from EHRs

Column	Type	Description
**Pid**	varchar(32)	Patient identifier
**Sample**	varchar(64)	Biosample identifier
**Type**	varchar(32)	Parameter (e.g., ECOG performance status)
**Value**	varchar(128)	Value (e.g., 1)
**documentation_date**	timestamp	Date of documentation

Supplementary Table 8: Treatment data from EHRs

Column	Type	Description
**Pid**	varchar(32)	Patient identifier
**Sample**	varchar(64)	Biosample identifier
**Therapy**	varchar(256)	Free text description of treatment
**therapy_start**	timestamp	Start of treatment
**therapy_end**	timestamp	End of treatment
**Cycles**	varchar(256)	Treatment cycles
**Response**	varchar(256)	Treatment response
**best_response**	varchar(256)	Best treatment response
**best_response_start**	timestamp	Start of best treatment response
**best_response_end**	timestamp	End of best treatment response

Supplementary Table 9: Tumor sample data from EHRs

Column	Type	Description
**Sample**	varchar(64)	Biosample identifier
**Pid**	varchar(32)	Patient identifier
**Sequencing**	varchar(64)	Sequencing method (e.g., WGS)
**Sequenced**	boolean	Availability of sequencing data available (yes or no)
**documentation_date**	timestamp	Date of documentation

Supplementary Table 10: Integrated biomarkers data from bioinformatics workflow

Column	Type	Description
**pid**	varchar(32)	Patient identifier
**sample**	varchar(64)	Biosample identifier
**diagnosis**	varchar(256)	Free text description of diagnosis
**hrd**	integer	HRD score
**lst**	integer	LST score
**tai**	integer	TAI score
**snvs**	integer	Number of functional somatic SNVs
**msisensor**	numeric	MSIsensor score
**ploidy**	integer	Base ploidy where most segments are localized
**purity**	integer	Tumor cell content
**indels**	integer	Number of functional somatic indels
**signatures**	varchar(2048)	Mutational signatures found
**somatic**	varchar(2048)	Genes with somatic alterations relevant for inclusion in the NCT PMO-1603/TOP-ART trial (NCT03127215)
**germline**	varchar(2048)	Genes with germline alterations relevant for inclusion in the NCT PMO-1603/TOP-ART trial
**cellcycle**	varchar(2048)	Biomarkers used for the "Cell Cycle" basket
**ras**	varchar(2048)	Biomarkers used for the "RAF-MEK-ERK" basket
**rtk**	varchar(2048)	Biomarkers used for the "Tyrosine Kinase" basket
**developmental**	varchar(2048)	Biomarkers used for the "Developmental Pathways" basket
**mtor**	varchar(2048)	Biomarkers used for the "PI3K-AKT-mTOR" basket
**dnarepair**	varchar(2048)	Biomarkers used for the "DNA Damage Response" basket
**immunotherapy**	varchar(2048)	Biomarkers used for the "Immune Evasion" basket
**other**	varchar(2048)	Biomarkers used for the "Other"
**sequencing**	varchar(256)	Sequencing method (e.g., WGS)
**tmb**	numeric	Tumor mutational burden
**tmbhigh**	numeric	Cut-off for the definition of high tumor mutational burden (unit: non-synonymous mutations per coding megabase)
**telomerecontent**	numeric	Telomere content ratio
**altscore**	numeric	Alternative lengthening of telomeres score
**baskets_geneswithhits**	varchar(256)	List of genes from the DKFZ/NCT/DKTK MASTER baskets with alterations
**topart_geneswithhits**	varchar(256)	List of genes with alterations relevant for inclusion in the NCT PMO-1603/TOP-ART trial
**quality**	varchar(256)	Quality of RNA-seq data
**documentation_date**	timestamp	Date of documentation

Supplementary Table 11: Mutational signatures data from bioinformatics workflow

Column	Type	Description
**pid**	varchar(32)	Patient identifier
**sample**	varchar(64)	Biosample identifier
**mutational_signature _sample**	varchar(64)	Set of mutational signatures (Valid: validated signatures; Artif: including artifact signatures) and algorithm (gen: general; abs: absolute, used for WGS; norm: normalized, used for WES)
**mutational_signature**	varchar(16)	Mutational signature
**normalised_upper _border**	numeric	Upper border of normalized exposure
**normalised_lower _border**	numeric	Lower border of normalized exposure
**relative_upper_border**	numeric	Relative upper border of exposure
**relative_lower_border**	numeric	Relative lower border of exposure
**normalised_exposure**	numeric	Normalized exposure
**exposure**	numeric	Exposure
**upper_border**	numeric	Upper border of exposure
**lower_border**	numeric	Lower border of exposure
**documentation_date**	timestamp	Date of documentation

Supplementary Table 12: HLA typing data from bioinformatics workflow

Column	Type	Description
**pid**	varchar(32)	Patient identifier
**sample**	varchar(64)	Biosample identifier
**hla_locus**	varchar(64)	HLA gene
**hla_sample_name**	varchar(64)	Sample type the HLA type was called from (control, tumor; DNA, RNA)
**hla_sample_value**	varchar(64)	HLA allele
**documentation_date**	timestamp	Date of documentation

Supplementary Table 13: Methylation classifier data from bioinformatics workflow

Column	Type	Description
**pid**	varchar(32)	Patient identifier
**sample**	varchar(64)	Biosample identifier
**type**	varchar(64)	Classifier type
**version**	varchar(64)	Version of classifier type
**prediction**	varchar(256)	Code of predicted diagnosis
**probability**	numeric	Probability of matching diagnosis
**diagnosis**	varchar(256)	Full text description of predicted diagnosis
**description**	varchar(2048)	Description of diagnosis cluster
**documentation_date**	timestamp	Date of documentation

Supplementary Table 14: Somatic SNV data from the somatic SNV calling workflow

Column	Type	Description
**pid**	varchar(32)	Patient identifier
**sample**	varchar(64)	Biosample identifier
**chromosome**	varchar(128)	Chromosome
**position**	integer	1-based position of variant
**dbsnp**	varchar(4096)	dbSNP rs identifier
**base_ref**	varchar(256)	Reference allele
**base_alt**	varchar(256)	Alternative allele
**confidence**	integer	Quality score associated with alleles inferred
**filter**	varchar(256)	Flag indicating which filters variant has failed or PASS if all filters were passed
**genes**	varchar(128)	HUGO gene symbols
**tumor_dna_af**	numeric	Variant allele frequency (tumor DNA)
**tumor_rna_af**	numeric	Variant allele frequency (tumor RNA)
**control_dna_af**	numeric	Variant allele frequency (control DNA)
**exonic_classification**	varchar(256)	Impact of variant on protein translation
**reclassification**	varchar(256)	Classification of variant as somatic or germline
**annovar_transcripts**	varchar(4096)	Information on transcripts annotated by ANNOVAR
**annovar_function**	varchar(4096)	Location of variant (intron, exon, splice site, untranslated region, upstream, downstream)
**ref_genome**	varchar(16)	Reference genome used
**documentation_date**	timestamp	Date of documentation

Supplementary Table 15: Germline SNV data from the somatic SNV calling workflow

Column	Type	Description
**pid**	varchar(32)	Patient identifier
**sample**	varchar(64)	Biosample identifier
**chromosome**	varchar(128)	Chromosome
**position**	integer	1-based position of variant
**dbsnp**	varchar(4096)	dbSNP rs identifier
**base_ref**	varchar(256)	Reference allele
**base_alt**	varchar(256)	Alternative allele
**confidence**	integer	Quality score associated with alleles inferred
**filter**	varchar(256)	Flag indicating which filters variant has failed or PASS if all filters were passed
**genes**	varchar(128)	HUGO gene symbols
**tumor_dna_af**	numeric	Variant allele frequency (tumor DNA)
**tumor_rna_af**	numeric	Variant allele frequency (tumor RNA)
**control_dna_af**	numeric	Variant allele frequency (control DNA)
**exonic_classification**	varchar(256)	Impact of variant on protein translation
**reclassification**	varchar(256)	Classification of variant as somatic or germline
**annovar_transcripts**	varchar(4096)	Information on transcripts annotated by ANNOVAR
**annovar_function**	varchar(4096)	Location of variant (intron, exon, splice site, untranslated region, upstream, downstream)
**ref_genome**	varchar(16)	Reference genome used
**documentation_date**	timestamp	Date of documentation

Supplementary Table 16: Somatic indel data from the somatic indel calling workflow

Column	Type	Description
**pid**	varchar(32)	Patient identifier
**sample**	varchar(64)	Biosample identifier
**chromosome**	varchar(128)	Chromosome
**position**	integer	1-based position of variant
**dbsnp**	varchar(4096)	dbSNP rs identifier
**base_ref**	varchar(256)	Reference allele
**base_alt**	varchar(256)	Alternative allele
**confidence**	integer	Quality score associated with alleles inferred
**filter**	varchar(256)	Flag indicating which filters variant has failed or PASS if all filters were passed
**genes**	varchar(128)	HUGO gene symbols
**tumor_dna_af**	numeric	Variant allele frequency (tumor DNA)
**tumor_rna_af**	numeric	Variant allele frequency (tumor RNA)
**control_dna_af**	numeric	Variant allele frequency (control DNA)
**exonic_classification**	varchar(256)	Impact of variant on protein translation
**reclassification**	varchar(256)	Classification of variant as somatic or germline
**annovar_transcripts**	varchar(4096)	Information on transcripts annotated by ANNOVAR
**annovar_function**	varchar(4096)	Location of variant (intron, exon, splice site, untranslated region, upstream, downstream)
**ref_genome**	varchar(16)	Reference genome used
**documentation_date**	timestamp	Date of documentation

Supplementary Table 17: Germline indel data from the somatic indel calling workflow

Column	Type	Description
**pid**	varchar(32)	Patient identifier
**sample**	varchar(64)	Biosample identifier
**chromosome**	varchar(128)	Chromosome
**position**	integer	1-based position of variant
**dbsnp**	varchar(4096)	dbSNP rs identifier
**base_ref**	varchar(256)	Reference allele
**base_alt**	varchar(256)	Alternative allele
**confidence**	integer	Quality score associated with alleles inferred
**filter**	varchar(256)	Flag indicating which filters variant has failed or PASS if all filters were passed
**genes**	varchar(128)	HUGO gene symbols
**tumor_dna_af**	numeric	Variant allele frequency (tumor DNA)
**tumor_rna_af**	numeric	Variant allele frequency (tumor RNA)
**control_dna_af**	numeric	Variant allele frequency (control DNA)
**exonic_classification**	varchar(256)	Impact of variant on protein translation
**reclassification**	varchar(256)	Classification of variant as somatic or germline
**annovar_transcripts**	varchar(4096)	Information on transcripts annotated by ANNOVAR
**annovar_function**	varchar(4096)	Location of variant (intron, exon, splice site, untranslated region, upstream, downstream)
**ref_genome**	varchar(16)	Reference genome used
**documentation_date**	timestamp	Date of documentation

Supplementary Table 18: Germline small variant (i.e., SNV and indel) data from the germline variant calling workflow

Column	Type	Description
**pid**	varchar(32)	Patient identifier
**sample**	varchar(64)	Biosample identifier
**chromosome**	varchar(128)	Chromosome
**position**	integer	1-based position of variant
**base_ref**	varchar(256)	Reference allele
**base_alt**	varchar(256)	Alternative allele
**filter**	varchar(256)	Flag indicating which filters variant has failed or PASS if all filters were passed
**genes**	varchar(128)	HUGO gene symbols
**transcript**	varchar(256)	Ensembl transcript ID
**variant_on_gene**	varchar(256)	Effect of variant on cDNA according to HGVSc
**variant_on_protein**	varchar(256)	Effect of variant on protein according to HGVSp
**rna_variant_expression**	varchar(256)	Text string indicating if variant was expressed (tumor RNA)
**tumor_dna_af**	numeric	Variant allele frequency (tumor DNA)
**tumor_rna_af**	numeric	Variant allele frequency (tumor RNA)
**control_dna_af**	numeric	Variant allele frequency (control DNA)
**max_gnom_ad_af**	varchar(256)	Maximum minor allele frequency in GnomAD
**max_gnom_ad_ac**	varchar(256)	Maximum count of individuals with minor allele in GnomAD
**max_gnom_ad_homo**	varchar(256)	Maximum count of individuals carrying the alternative/minor allele homozygously in GnomAD
**max_lc_vf**	varchar(256)	Maximum variant frequency in local control WGS and WES data
**vep_most_ severe_consequence**	varchar(256)	Most severe consequence of variant on coding sequence according to Variant Effect Predictor
**all_functional_consensus**	varchar(1028)	Consensus functional consequence on coding sequence
**cadd_phred**	varchar(256)	Variant pathogenicity according to CADD score on PHRED scale
**acmg_classification**	varchar(256)	Pathogenicity according to ACMG criteria
**variant_classification**	varchar(256)	Impact of variant on protein translation
**impact**	varchar(256)	Predicted impact of variant
**charger_score**	varchar(256)	Pathogenicity of variant calculated with CharGer
**charger_summary**	varchar(4096)	Pathogenicity of variant calculated with CharGer
**charger_classification**	varchar(256)	Pathogenicity of variant calculated with CharGer
**clinvar_pathogenicity**	varchar(256)	Pathogenicity of variant according to ClinVar
**clinvar_traits**	varchar(4096)	Traits associated with variant in ClinVar
**clnid**	varchar(256)	ClinVar identifier
**cln_inclusion_status**	varchar(256)	Flag if variant is present in ClinVar inclusion list
**clnrevstat**	varchar(256)	Review status of variant in ClinVar
**clnsig**	varchar(256)	Pathogenicity of variant in ClinVar
**clnsigconf**	varchar(256)	Confidence of variant pathogenicity in ClinVar
**hgnc_url**	varchar(256)	Link to gene entry in HGNC
**hgvsg_exon_intron**	varchar(256)	Number of variant-containing exon or intron
**splice_ai_ds_gt_04**	varchar(256)	Predicted effect of variant on splicing according to SpliceAI with high recall and low precision (0.4)
**splice_ai_ds_gt_09**	varchar(256)	Predicted effect of variant on splicing according to SpliceAI with low recall and high precision (0.9)
**tumor_sample**	varchar(256)	Tumor sample used for variant calling
**ref_genome**	varchar(16)	Reference genome used
**documentation_date**	timestamp	Date of documentation

Supplementary Table 19: CNV data (WES) from bioinformatics workflow

Column	Type	Description
**pid**	varchar(32)	Patient identifier
**sample**	varchar(64)	Biosample identifier
**cnv_type**	varchar(32)	Type of CNV
**chromosome**	varchar(128)	Chromosome
**start**	integer	Start of CNV
**end**	integer	End of CNV
**length**	integer	Length of CNV
**genes**	varchar(128)	Genes affected by CNV
**tcn**	numeric	Total copy number
**ploidy**	integer	Base ploidy where most segments are localized
**ref_genome**	varchar(16)	Reference genome used
**documentation_date**	timestamp	Date of documentation

Supplementary Table 20: CNV data (WGS) from bioinformatics workflow

Column	Type	Description
**pid**	varchar(32)	Patient identifier
**sample**	varchar(64)	Biosample identifier
**cnv_type**	varchar(32)	Type of CNV
**chromosome**	varchar(128)	Chromosome
**start**	integer	Start of CNV
**end**	integer	End of CNV
**length**	integer	Length of CNV
**genes**	varchar(128)	Genes affected by CNV
**bp_genes**	varchar(4096)	Indication if gene located at breakpoint
**tcn**	numeric	Total copy number
**ploidy**	integer	Base ploidy where most segments are localized
**ref_genome**	varchar(16)	Reference genome used
**documentation_date**	timestamp	Date of documentation

Supplementary Table 21: SV data from bioinformatics workflow

Column	Type	Description
**pid**	varchar(32)	Patient identifier
**sample**	varchar(64)	Biosample identifier
**sv_type**	varchar(32)	Type of SV
**chromosome_1**	varchar(128)	Information on first chromosome
**chromosome_2**	varchar(128)	Information on second chromosome
**position_1**	integer	Position of breakpoint on first chromosome
**position_2**	integer	Position of breakpoint on second chromosome
**event_size**	integer	Distance between breakpoints
**event_score**	integer	Score from 1 to 5 indicating reliability of SV call
**genes_1**	varchar(128)	HUGO gene symbols
**genes_2**	varchar(128)	HUGO gene symbols
**direct_fusion _candidates**	varchar(4096)	Potential fusions between genes affected
**ref_genome**	varchar(16)	Reference genome used
**documentation_date**	timestamp	Date of documentation

Supplementary Table 22: Fusion data from bioinformatics workflow

Column	Type	Description
**pid**	varchar(32)	Patient identifier
**sample**	varchar(64)	Biosample identifier
**pipeline**	varchar(64)	Fusion calling pipeline used
**type**	varchar(128)	Type of fusion
**confidence**	varchar(64)	Confidence of fusion call
**filters**	varchar(256)	Filters used to remove supporting reads
**chromosome_1**	varchar(128)	Information on first chromosome
**chromosome_2**	varchar(128)	Information on second chromosome
**breakpoint_1**	integer	Position of breakpoint on first chromosome
**breakpoint_2**	integer	Position of breakpoint on second chromosome
**closest_genomic _breakpoint_1**	varchar	DNA breakpoint closest to first RNA breakpoint
**closest_genomic _breakpoint_2**	varchar	DNA breakpoint closest to second RNA breakpoint
**coverage_1**	integer	Number of reads at first breakpoint
**coverage_2**	integer	Number of reads at second breakpoint
**direction_1**	varchar(64)	Direction of reads from first fusion partner
**direction_2**	varchar(64)	Direction of reads from second fusion partner
**site_1**	varchar(128)	Position of functional sites at first breakpoint
**site_2**	varchar(128)	Position of functional sites at second breakpoint
**split_reads_1**	integer	Number of split reads supporting gene 1
**split_reads_2**	integer	Number of split reads supporting gene 2
**strand_1**	varchar(8)	Transcribed strand for gene 1
**strand_2**	varchar(8)	Transcribed strand for gene 2
**genes_1**	varchar(128)	Gene at fusion N-terminus
**genes_2**	varchar(128)	Gene at fusion C-terminus
**transcript_id_1**	varchar(128)	Ensembl transcript identifier for gene 1
**transcript_id_2**	varchar(128)	Ensembl transcript identifier for gene 2
**fusion_transcript**	varchar(2058)	Fusion transcript sequence
**discordant_mates**	integer	Number of read pairs of discordant mates supporting fusion
**peptide_sequence**	varchar(2058)	Fusion peptide sequence
**read_identifiers**	varchar(2058)	Names of supporting reads
**reading_frame**	varchar(128)	Reading frame of fusion product
**retained_proteine _domains**	varchar(2058)	Protein domains retained by fusion product
**tags**	varchar(128)	User-defined tags for positions of interest
**ref_genome**	varchar(16)	Reference genome used
**documentation_date**	timestamp	Date of documentation

Supplementary Table 23: Gene expression data from bioinformatics workflow

Column	Type	Description
**pid**	varchar(32)	Patient identifier
**sample**	varchar(64)	Biosample identifier
**gene**	varchar(32)	Gene name
**expression**	numeric	Expression value (e.g., RPKM, FPKM, TPM)
**fold_change**	numeric	Fold-change expression compared to median of reference cohort
**z_score**	numeric	z-score of expression value compared to reference cohort
**rpkm_rank**	integer	Rank of expression compared to reference cohort
**reference_cohort**	varchar(16)	Name of reference cohort
**ref_genome**	varchar(16)	Reference genome used
**documentation_date**	timestamp	Date of documentation

Supplementary Table 24: Multibitmap files from bioinformatics workflow

Column	Type	Description
**pid**	varchar(32)	Patient identifier
**sample**	varchar(64)	Biosample identifier
**file_data**	bytea	Binary data of any kind (e.g., images, documents)
**file_name**	varchar(1024)	File name
**file_type**	varchar(64)	File data type
**file_date**	timestamp	File date
**file_size**	numeric	File size
**documentation_date**	timestamp	Date of file upload

Supplementary Table 25: Pharmacogenomics data from bioinformatics workflow

Column	Type	Description
**pid**	varchar(32)	Patient identifier
**sample**	varchar(64)	Biosample identifier
**gene**	varchar(256)	HUGO gene symbols
**harmonized_genotype**	varchar(256)	Consensus genotype from harmonization of genotypes from all tools
**harmonized_phenotype**	varchar(256)	Translated phenotype based on harmonized genotype
**pharmacogenomic_sample**	varchar(256)	Biosample identifier used by pharmacogenomics pipeline
**stargazer_genotype**	varchar(256)	Raw genotype output from Stargazer
**stargazer_curated_genotype**	varchar(256)	Curated and formatted genotype from Stargazer
**stargazer_genotype_tags**	varchar(256)	Tags with additional information on alleles in Stargazer genotype
**aldy_genotype**	varchar(256)	Raw genotype output from aldy
**aldy_curated_genotype**	varchar(256)	Curated and formatted genotype from Aldy
**aldy_genotype_tags**	varchar(256)	Tags with additional information on alleles in Aldy genotype
**pypgx_genotype**	varchar(256)	Raw genotype output from PyPGx
**pypgx_curated_genotype**	varchar(256)	Curated and formatted genotype from PyPGx
**pypgx_genotype_tags**	varchar(256)	Tags with additional information on alleles in PyPGx genotype
**cyrius_genotype**	varchar(256)	Raw genotype output from Cyrius
**cyrius_curated_genotype**	varchar(256)	Curated and formatted genotype from Cyrius
**cyrius_genotype_tags**	varchar(256)	Tags with additional information on alleles in Cyrius genotype
**stargazer_phenotype**	varchar(256)	Translated phenotype based on curated Stargazer genotype
**pypgx_phenotype**	varchar(256)	Translated phenotype based on curated PyPGx genotype
**stargazer_dip_sv**	varchar(256)	Additional Stargazer CNV information
**pypgx_cnv**	varchar(256)	Additional PyPGx CNV information
**harmonization_comment**	varchar(256)	Comments from harmonization process regarding special cases
**harm_allele1**	varchar(256)	Allele 1 of harmonized genotype
**harm_allele2**	varchar(256)	Allele 2 of harmonized genotype
**documentation_date**	timestamp	Date of documentation

Supplementary Table 26: Pharmacogenomics recommendations from bioinformatics workflow

Column	Type	Description
**pid**	varchar(32)	Patient identifier
**sample**	varchar(64)	Biosample identifier
**gene**	varchar(256)	HUGO gene symbols
**harmonized_genotype**	varchar(256)	Consensus genotype from harmonization of genotypes from all tools
**harmonized_phenotype**	varchar(256)	Translated phenotype based on harmonized genotype
**pharmacogenomic _sample**	varchar(256)	Biosample identifier used by the pharmacogenomics pipeline
**drug**	varchar(1028)	Drug
**recommendation**	varchar(4096)	Free text of recommendation provided by knowledge base
**knowledgebase**	varchar(256)	Underlying knowledge base (e.g., CPIC)
**documentation_date**	timestamp	Date of documentation

Supplementary Table 27: Knowledge bases selected for content relevant to clinical decision-making

Knowledge base	Queries	Transcripts	Oncogenicity	Biological effect	Drugs and therapies	Pathways	Gene description
CIViC[A]	Gene, variant				X		X
JAX-CKB[B,C]	Gene, variant		X	X	X		X
Ensembl[A]	Gene	X					
OncoKB[A]	Gene, variant		X	X	X		
Reactome[A]	Gene					X	

[A] Publicly available knowledge base
[B] Commercial knowledge base
[C] BoCKs from JAX-CKB are not part of the public KC instance.
